# Clonal Analysis of *kit ligand a* Functional Expression Reveals Lineage-Specific Competence to Promote Melanocyte Rescue in the Mutant Regenerating Caudal Fin

**DOI:** 10.1371/journal.pone.0102317

**Published:** 2014-07-10

**Authors:** Robert C. Tryon, Stephen L. Johnson

**Affiliations:** Washington University School of Medicine, Department of Genetics, St. Louis, Missouri, United States of America; Deakin School of Medicine, Australia

## Abstract

The study of regeneration in an *in vivo* vertebrate system has the potential to reveal targetable genes and pathways that could improve our ability to heal and repair damaged tissue. We have developed a system for clonal labeling of discrete cell lineages and independently inducing gene expression under control of the heat shock promoter in the zebrafish caudal fin. Consequently we are able to test the affects of overexpressing a single gene in the context of regeneration within each of the nine different cell lineage classes that comprise the caudal fin. This can test which lineage is necessary or sufficient to provide gene function. As a first example to demonstrate this approach, we explored which lineages were competent to functionally express the *kit ligand a* protein as assessed by the local complementation of the mutation in the *sparse-like* (*kitlga* ^tc244b^) background. We show that dermal fibroblast expression of *kit ligand a* robustly supports the rescue of melanocytes in the regenerating caudal fin. *kit ligand a* expression from skin and osteoblasts results in more modest and variable rescue of melanocytes, while lateral line expression was unable to complement the mutation.

## Introduction

Understanding the roles that individual genes play in different cell lineages during the regeneration of injured tissue remains a significant challenge for developmental biologists and regenerative medicine. Regeneration of particular cells or lineages involves a variety of cues and signals. These may be either autonomous to the cells in question or may be provided from neighboring cells. In order to begin to probe the genes and pathways required for the regeneration of distinct cell lineages, we set out to develop an experimental system that would allow us to perturb or induce expression of a gene within defined cells or lineages in vivo. The zebrafish fin is ideally suited for this approach [1]–[12].

Previous work in our lab has demonstrated that transposon-labeled fin clones, when challenged to regenerate, faithfully reproduce the same cell type [13]. Other research groups using alternative approaches to label clones also failed to see any transdifferentiation between tissues in the regenerating caudal fin [14]–[15]. We reasoned we could use this transposon system to manipulate gene expression under the control of a heat shock promoter, hsp70l [16]. This promoter drives heat shock dependent gene expression in all of the lineages of the regenerating fin (not shown) [17]–[18]. When the transposon also harbors a lineage tracer the clone can be unambiguously identified and interrogated for the consequence of altered gene expression in each of the nine lineage classes. This construct is randomly integrated into fin lineage progenitors during embryonic stages using the Tol2 transposon. When injected animals grow to adult stages they will bear mosaic fins that can be easily screened and manipulated. Consequently we can ask how a gene acts in each of the fin lineages classes in the context of regeneration.

As a first demonstration of this approach of clonal gene manipulation analysis we set out to explore the role of *kit ligand a* (*kitlga*) signaling in the regenerating fin. *kitlga* (also known as stem cell factor), interacts with the *Kit* receptor tyrosine kinase, *kita*, and plays a key role in the development and homeostasis of zebrafish melanocytes [19]. A role for *kita* function has been shown in zebrafish melanocyte development both during early larval stages [20] as well as in the regenerating caudal fin [21]–[22]. Two more recent studies have implicated the interaction of *kita* and *kitlga* in melanocyte stem cell development [23]–[24].

Following caudal fin amputation, the melanocytes regenerate from a melanocyte stem cell population [25], ultimately reconstituting the stereotypical stripe pattern of the zebrafish. Experiments using *kita* mutants have demonstrated an initial period of ∼7 days in which *kita* function is needed to support melanocyte regeneration [21]–[22]. These *kit*-dependent melanocytes differentiate in distal (younger) locations in the regenerate. After 7 days, *kit*-independent melanocytes begin to differentiate in proximal positions, ultimately reconstituting the entire stripe. We show here that *kitlga* has an identical fin regeneration phenotype as the *kita* mutant. In contrast to *kita*, which acts autonomously in the melanocyte, it is not clear which cell type can confer productive *kitlga* activity. We reasoned transposon-based clonal gene manipulation would serve as an ideal way to explore this question. We chose to randomly replace *kitlga* expression in different cell lineages of the *sparse-like* mutant[24], hereafter referred to as *kitlga* ^tc244b^, to assay for rescue of *kit*-dependent melanocytes.

We report the use of the pT2-hsp70l transposon for use in clonal analysis of fin clones. This transposon allows us to separately label each of the cell types that make up the fish fin with GFP, and in this set of experiments drive expression of *kitlga* within these clones when subjected to a ∼1hr heat shock (38°C). We find that dermal fibroblasts robustly support melanocyte rescue in the regenerating fish fin. In contrast, skin and osteoblast clones were more variable in their ability to support melanocyte regeneration. In addition we found that skin clones showed some regional differences in their ability to promote melanocyte rescue. Specifically, skin clones were less competent to support melanocyte regeneration when the clone fell within a xanthophore stripe.

## Materials and Methods

### Generating Clones

**Figure 1:**
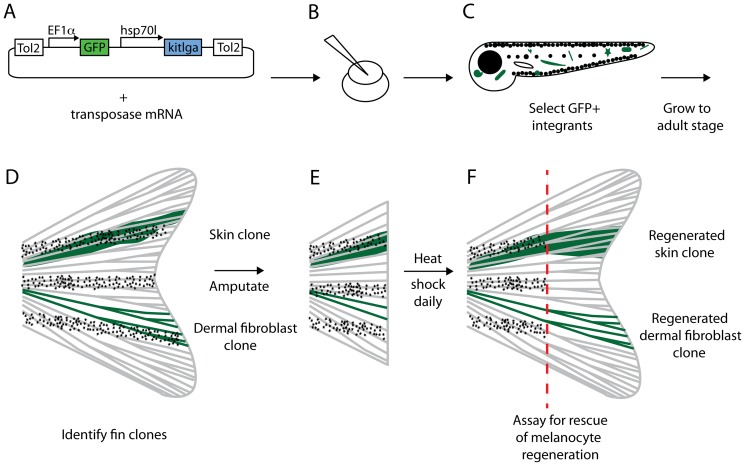
Experimental procedure for clonal gene expression analysis of *kitlga.* A. The pt2-hsp70l>*kitlga* transposon consists of a *Xenopus* ef1a promoter driving GFP for labeling and identification of clone lineages and a *Danio rerio* hsp70l promoter driving *kitlga* to allow heat inducible expression of *kitlga.* B. Clones are generated by co-injecting pT2-hsp70l>*kitlga* DNA with transposase mRNA into 1 cell *sparse-like* (*kitlga* ^tc244b^) embryos. C. Larvae are screened initially at 3dpf to select for GFP+ integrants. D. GFP+ larvae are subsequently grown to adult stages when they are re-screened for caudal fin clones, shown in green. E. Fins are amputated, leaving behind labeled cells from the clone in the stump to regenerate Fish are then placed in a heat shock cabinet in order to provide daily heat shock pulses of 38°C. F. After 7 days of regeneration and heat shock induction of *kitlga*, fins are observed for rescue of melanocyte regeneration.

To generate clones expressing *kitlga* protein in *kitlga* ^tc244b^ mutant fish we took advantage of the pt2-hsp70l>*kitlga* transposon [26]. The two key components of the pT2-hsp70l>*kitlga* construct are (1) the Xenopus ef1α>GFP cassette which labels the clone and (2) the Danio rerio hsp70l>*kitlga* cassette allowing for heat inducible expression of *kitlga cDNA (*NM_001018123) (Figure 1A). *kitlga* ^tc244b^ mutant embryos were generated by in vitro fertilization and injected at the 1-cell stage with 5 nL of a mixture of pT2-hsp70l>*kitlga* (15 ng/µL) and transposase mRNA (5 ng/µL) (Figure 1B).

### Clone Identification

Clones were identified at two stages. First we screened injected larvae at 3 days post fertilization (dpf) for any GFP expression, indicating one or more successful integrations of the transposon (Figure 1C). While most integration events of the transposon occur around the 4,000 cell stage, subsequent growth and development mix such integrated cell lineages throughout the embryo [27]. Since we could not tell at 3dpf whether the GFP+ integrated cells would ultimately establish clones in the caudal fin, we grew the fish to adult stages. At 12 weeks of age, fish were re-screened to identify GFP+ clones within the caudal fin (Figure 1D). Fish with fin clones were grouped according to tissue type and photographed. Fish were placed in a 40 mg/L solution of tricaine methanesulfonate (Western Chemical Inc., Ferndale, WA) to anesthetize fish and a portion of their caudal fin was amputated with a sharp razor blade (Figure 1E). The proximal portion of the clone was left intact in the stump to allow for the regeneration of labeled cells. Following amputation, fish were either returned to our standard system (25°C) as controls or placed on a modified Marine Biotech Z-mod system at a density of 5 fish per liter, where they received daily 1-hour heat shock exposures of 38°C to induce *kitlga* expression [28]–[29]. Regenerated fins were observed and photographed at 7 days post amputation (dpa) (Figure 1F).

### Heat Shock Induction

A Marine Biotech Z-mod cabinet was modified to create a reliable daily 1 hour heat shock pulse of 38°C [28]–[29]. To allow for a sufficiently quick ramping of water temperature in the system, the total amount of circulating water was reduced to ∼72L. 2 large heater units with a combined wattage of 1350 W were placed at the bottom of the sump and activated for 2 hours, 20 minutes each morning with an electric timer. This allowed a gradual increase in water temperature for delivering a heat shock pulse and minimized observable stress to adult fish. Fresh water was constantly added to the system at an influx rate of 2.4L per hour to help cool the water back to 25°C following heat shock.

### Animal Care

This study was carried out in strict accordance with the recommendations in the Guide for the Care and Use of Laboratory Animals of the National Institutes of Health. The protocol was approved by the Animal Studies Committee of Washington University in St. Louis (A-3381-01; 20110236). All caudal fin amputations were performed under tricaine methanesulfonate anesthetic.

## Results

**Figure 2:**
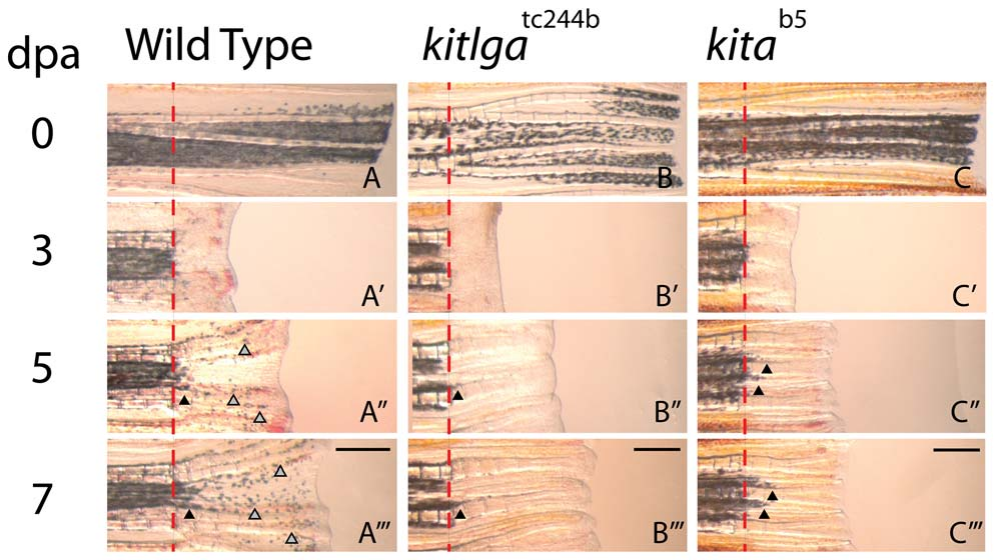
*sparse-like* (*kitlga* ^tc244b^) caudal fins fail to regenerate melanocytes during the initial 7 days post amputation (dpa). A, B, C. Wild-type, *sparse-like* (*kitlga* ^tc244b^), and sparse (*kita* ^b5^) fins were amputated and allowed to regenerate at 25°C. A’, B’, C’. No new melanocytes are observed during the first 3 dpa, irrespective of genotype. A’’. By 5 dpa, wild-type fins have newly differentiated *kit*-dependent melanocytes (grey arrowheads) in the regenerate. A’’’. By 7 dpa, more melanocytes emerge and organize into stripes. B’’-B’’’. *kitlga* ^tc244b^ fins fail to produce new melanocytes during the first 7 dpa. C’’-C’’’. *kita* ^b5^ fins fail to produce new melanocytes during the first 7 dpa. The few melanocytes near the amputation plane (dashed-line) in the regenerate of *kitlga* ^tc244b^ *and kita* ^b5^ fins are previously differentiated melanocytes (black arrowheads) that are drawn from the stump into the regenerated tissue as it grows. Scale bar  =  500 mm.

We first asked whether *kitlga* ^tc244b^ mutants showed the same melanocyte regeneration defects in the regenerating caudal fin as mutants with a defective *kita* receptor [21]. Wild type, *kitlga* ^tc244b^, and *kita* ^b5^ caudal fin regenerates were photographed during the 7 days following amputation (Fig 2). In the wild type fin, new faint melanocytes (grey arrowheads) can be seen by 5 dpa (Fig 2A’’), and continue to increase in number through 7dpa (Fig 2A’’’). In contrast, both *kitlga* ^tc244b^ and *kita* ^b5^ fins failed to develop any new melanocytes during this period. The small number of melanocytes (black arrowheads) immediately adjacent to the amputation plane (dashed line) within the regenerate of all 3 genotypes are previously differentiated, *kit*-independent melanocytes (Fig 2A’’-A’’’, 2B’’-2B’’’, 2C’’-2C’’’). As new tissue forms, a few previously differentiated melanocytes are drawn from the stump into the regenerate at its proximal boundary. There are no *kit*-dependent melanocytes in the distal regenerate of either *kitlga* ^tc244b^ and *kita* ^b5^ fins at 7 dpa. Accordingly we can use the presence of distal melanocytes at 7 dpa as evidence of *kitlga* promoted rescue.

**Figure 3:**
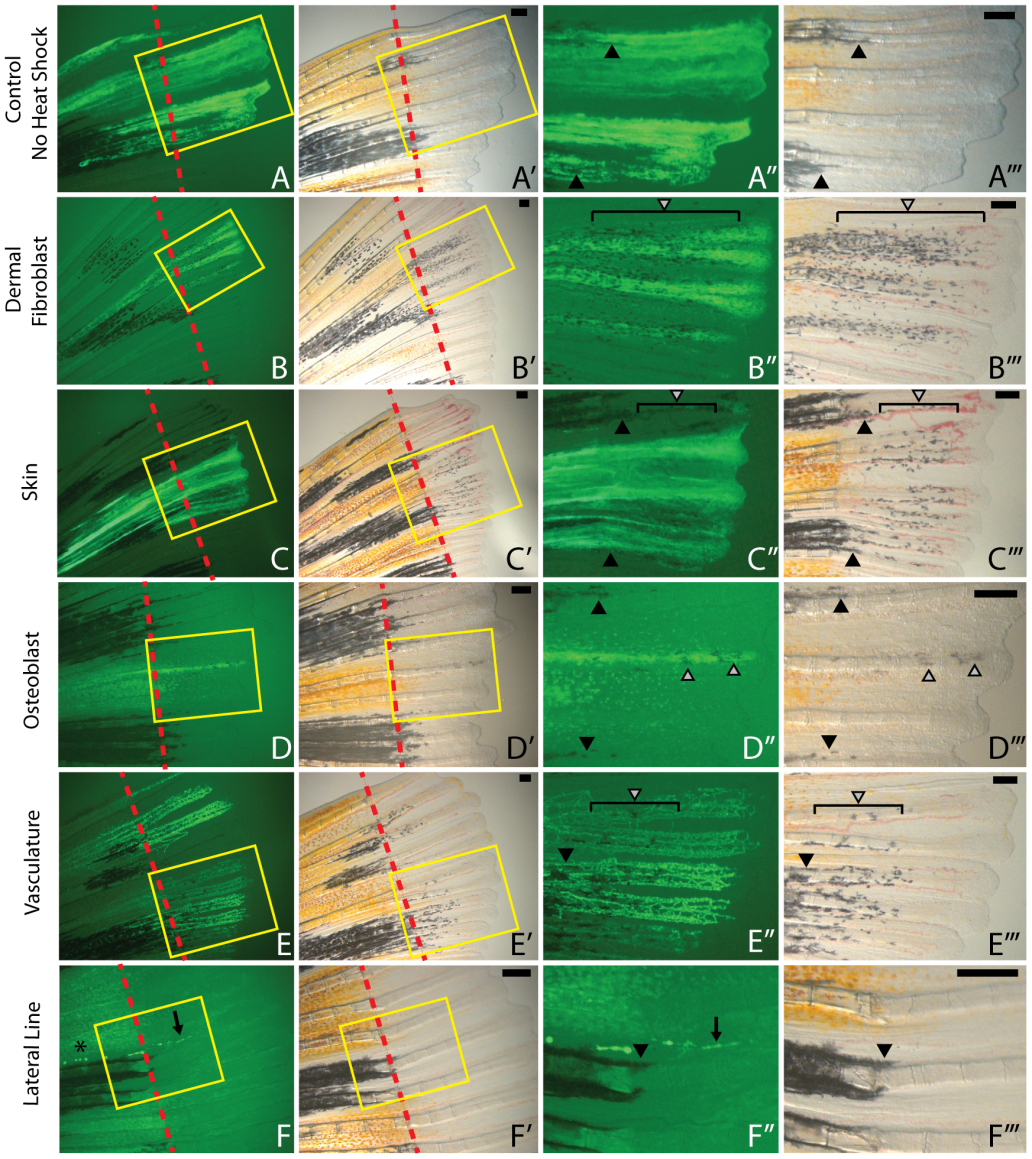
Multiple clone lineages are competent to support melanocyte rescue in the regenerating fins of *kitlga* ^tc244b^ mutants following expression of *kitlga*. A-A’’’. Skin clone that has not been subjected to heat shock shows no rescued melanocytes associated with the *kitlga* clone. B-B’’’. Dermal fibroblasts robustly support melanocyte rescue as a result of expressing *kitlga.* C-E. Skin (C-C’’’), osteoblasts (D-D’’’), and vasculature (E-E’’’) can support melanocyte rescue, but with a greater degree of variability of the strength of rescue as compared to dermal fibroblasts. F-F’’’. Lateral line clones (black arrow) could be distinguished from intra-ray glia by their neuromasts (asterisk) and were never able to rescue melanocytes. Strong rescue is shown in B’’’, C’’’, and E’’’. Weak rescue is shown in D’’’. Red dashed lines indicate the amputation plane. Yellow rectangles in A and A’ indicate insets magnified in greater detail in A’’ and A’’’, respectively. Grey arrowheads indicate newly differentiated *kit*-dependent melanocytes. Black arrowheads indicate previously differentiated melanocytes drawn into the regenerate from the stump. Scale bar  =  250 mm.

**Figure 4:**
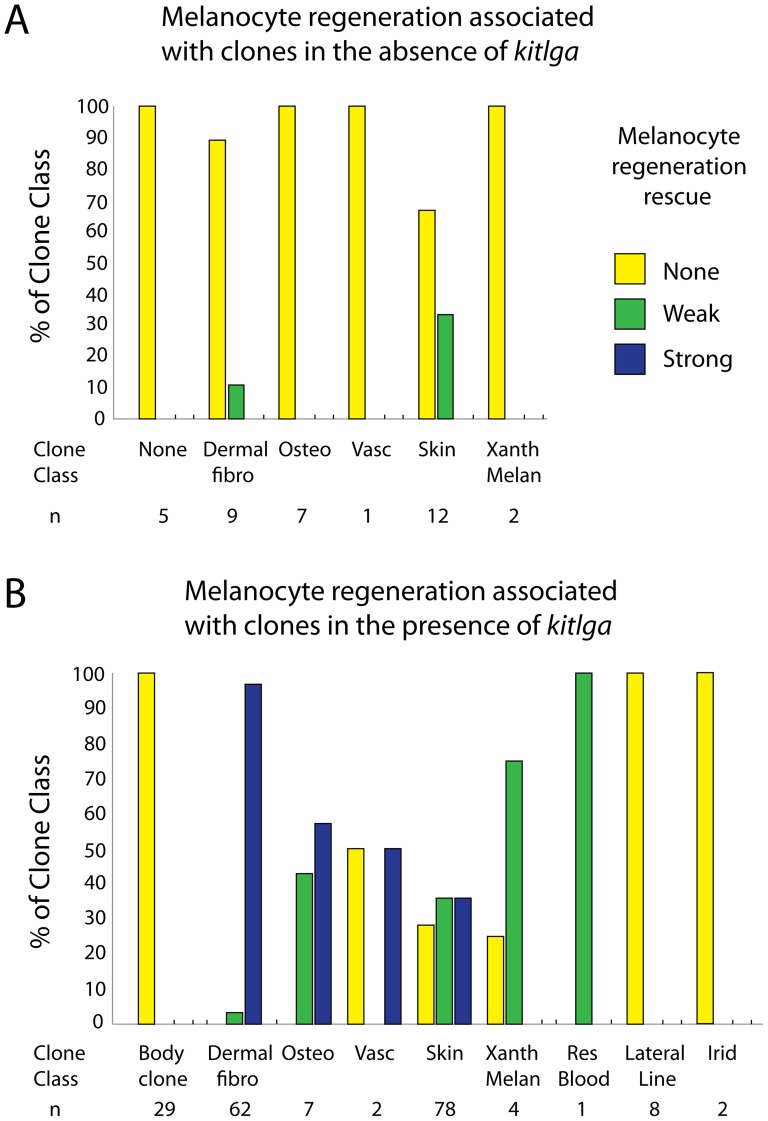
Summary of melanocyte rescue by pt2-hsp70l>*kitlga* clones. A. The majority of clones failed to rescue *kit*-dependent melanocytes in the absence of heat shock induced *kitlga* expression. Strong rescue was never observed and only 16% of uninduced clones showed weak rescue. B. Following heat shock induction of *kitlga*, variable levels of melanocyte rescue were observed depending on the labeled lineage. Dermal fibroblast showed the clearest effect of strongest rescue. Skin, osteoblast, vascualure, xanthophore/melanocyte, and resident blood showed more variable and weaker rescue. Lateral line and iridiphore clones were unable to rescue melanocytes during regeneration.

We first asked whether there was any evidence of uninduced expression of *kitlga* from the hsp70l promoter in the absence of a daily heat shock pulse. We observed little or no rescue in all assayed clone classes, including skin clones (Figure 3A-A’’’). In a few instances, skin clones (4/12, 33%) and a single dermal fibroblast clone (1/9, 11%) were associated with new melanocytes in the proximal regenerate in the absence of heat shock induced expression (Figure 4A). Since *kit*-independent melanocytes begin to differentiate in proximal positions after 7 days it is possible that these are precocious *kit*-independent melanocytes. Regardless, this provides a low level of background to compare our heat shock results.

Since most of the fish have additional *kitlga* clones in the body of the fish, separate from those found in the caudal fin, we needed to determine if distant expression would affect our assay. We saw no melanocyte rescue in the fin regenerate of fish with only body clones (n = 29) receiving daily heat shock exposure (Figure 4B). This indicates we can disregard *kitlga* expression from sources in the body that theoretically could produce circulating soluble ligand.

We next asked which lineage classes in the fin could support rescue of *kit*-dependant melanocytes following heat shock induction of *kitlga* expression. We found that dermal fibroblast (Fig 3 B-B’’’), skin (Fig 3C-C’’’), osteoblasts (Fig 3D-D’’’), vasculature (Fig 3E-E’’’), and xanthophore/melanocyte (not shown) clones could support melanocyte rescue through the expression of *kitlga*. Higher magnification imaging of osteoblast and dermal fibroblast clones make them distinguishable by their respective labeling of the outer sheath of the ray or the internal cell mass within the two hemirays [13, Fig 3E-J]. Notably we saw variability in the quality of melanocyte rescue in many of the clone classes, particulary skin (Figure S1). Representative strong melanocyte rescue is shown by dermal fibroblast (Fig 3B’’’), skin (Fig 3C’’’), and vasculature (Fig 3E’’’) clones while an example of weak rescue is shown by an osteoblast clone (Fig 3D’’’). One resident blood clone was able to weakly rescue melanocytes (not shown). Lateral line (Figure 3F-F’’’) and iridiphore (not shown) clonal expression of *kitlga* failed to rescue melanocytes. A single intra-ray glia clone was identified in our study, but this clone failed to regenerate following amputation. Consequently we were unable to ask if intra-ray glia expression of *kitlga* could support *kit*-dependent melanocyte rescue. The number of clones assayed with this protocol and the relative strengths of melanocyte rescue are described in Figure 4B. After 7 dpa *kita* (and *kitlga*)-independent mechanisms begin to contribute to melanocyte regeneration and therefore we do not report additional data beyond this time point.

**Figure 5:**
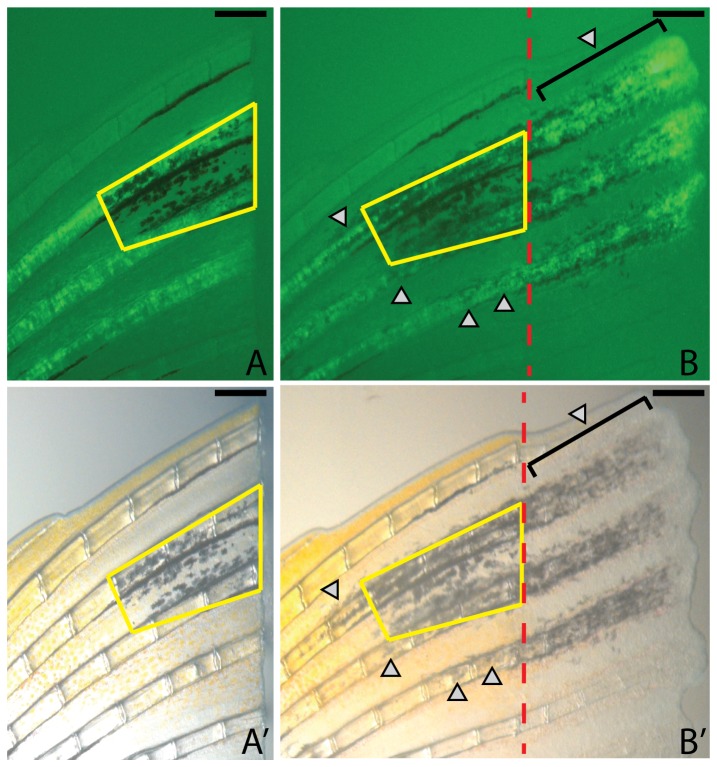
Expression of *kitlga* promotes differentiation of new melanocytes in the stump. A-A’. At the time of amputation and prior to induction of *kitlga*, melanocytes are well organized and form the proximal portion of the dorsal melanocyte stripe of the caudal fin (yellow trapezoid). B-B’. Following 7 days of *kitlga* expression, new melanocytes are visible both in the regenerate (brackets) as well as in the stump (grey arrowheads) in association with the dermal fibroblast clone. Red dashed lines indicate the amputation plane. Scale bar  =  250 mm.

In the proceeding analysis we asked whether *kitlga* could rescue *kit*-dependent melanocytes in the regenerating fin. Presumably the melanocyte stem cells are primed for *kitlga* activation in the regenerate. In contrast, stem cells in the stump are typically quiescent and may be refractory to *kitlga* induction. To explore this we looked at stumps within our study. An example of a dermal fibroblast clone that adds melanocytes in the stump during the heat shock induction of *kitlga* is shown in Fig 5. At the time of amputation (Fig 5A- A’), melanocytes form a well-organized stripe, despite an adjacent dermal fibroblast clone. After 7days of heat shock (Fig 5B-B’), melanocyte rescue is supported not only in the new distal regenerate (brackets), but also in the proximal stump (grey arrowheads) in locations where no melanocytes were detected previously. Similar to melanocytes in the regenerate, these new stump melanocytes are faint and small, supporting the notion that they are new melanocytes rather than older differentiated melanocytes that migrated toward the clone. Melanocytes rarely arise in the stump outside the stripe in fish without clones and without heat shock. Moreover, in a stable transgenic line expressing the pt2-hsp70l>*kitlga* construct (j996) heat shock is sufficient to induce new melanocytes outside the stripe without amputation (not shown).

**Figure 6:**
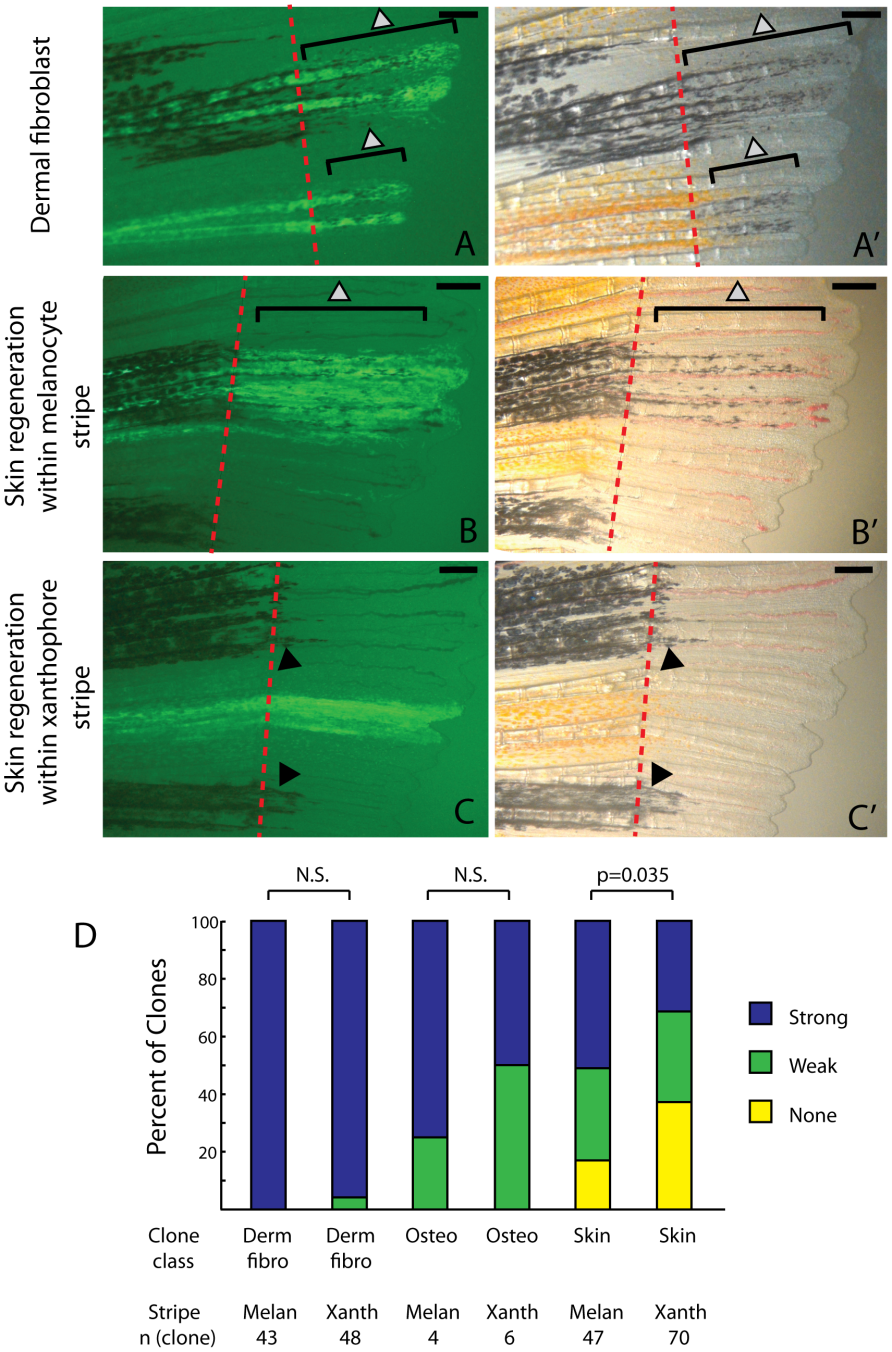
Skin clones show diminished ability to rescue melanocytes in xanthophores stripe regions of the regenerate. A-A’. Dermal fibroblasts strongly rescue melanocytes irrespective of where the clone occurs in the fin. B-C. Skin clones within the melanocyte stripe (B-B’) show more robust melanocyte rescue than skin clones that occur in a xanthophores stripe (C-C’). D. Dermal fibroblast, osteoblast, and skin clones were scored for the quality of melanocyte regeneration relative to their occurrence in a presumptive melanocyte stripe or xanthophore stripe. A single clone (as summarized in figure 4) may be scored as two clones if it occurs in both a xanthophore and melanocyte stripe region. Only skin clones showed a statistically significant difference in strength of rescue relative to the region in which the clone was regenerated (chi-squared 3×2 test, p value  = 0.035.) Red dashed lines indicate the amputation plane. Grey arrowheads indicate newly differentiated *kit*-dependent melanocytes. Black arrowheads indicate previously differentiated *kit*-independent melanocytes. Scale bar  =  250 mm.

Next we asked whether the clone’s regional position within the fin affects the ability of *kitlga* to rescue *kit*-dependent melanocytes. Even though *kit*-dependent melanocytes first develop unpatterned in the wild type regenerate (Figure 2A’’), *kitlga* might be more effective at signaling in presumptive melanocyte stripes as compared to presumptive xanthophore stripes. Dermal fibroblast clones strongly rescue melanocytes irrespective of their location relative to pre-existing stripes (Fig 6A-A’, 6D). In contrast, skin clones often showed better melanocyte rescue when the clone was in a melanocyte stripe region (Fig 6B-B’) as compared to being within a xanthophore stripe region (6C-C’). Subdividing all skin clones relative to their presence in a melanocyte or xanthophores stripe showed a small, but significant difference in their quality of melanocyte rescue via *kitlga* expression (Fig. 6D, chi-squared 3×2 test, p value  =  0.035).

**Figure 7:**
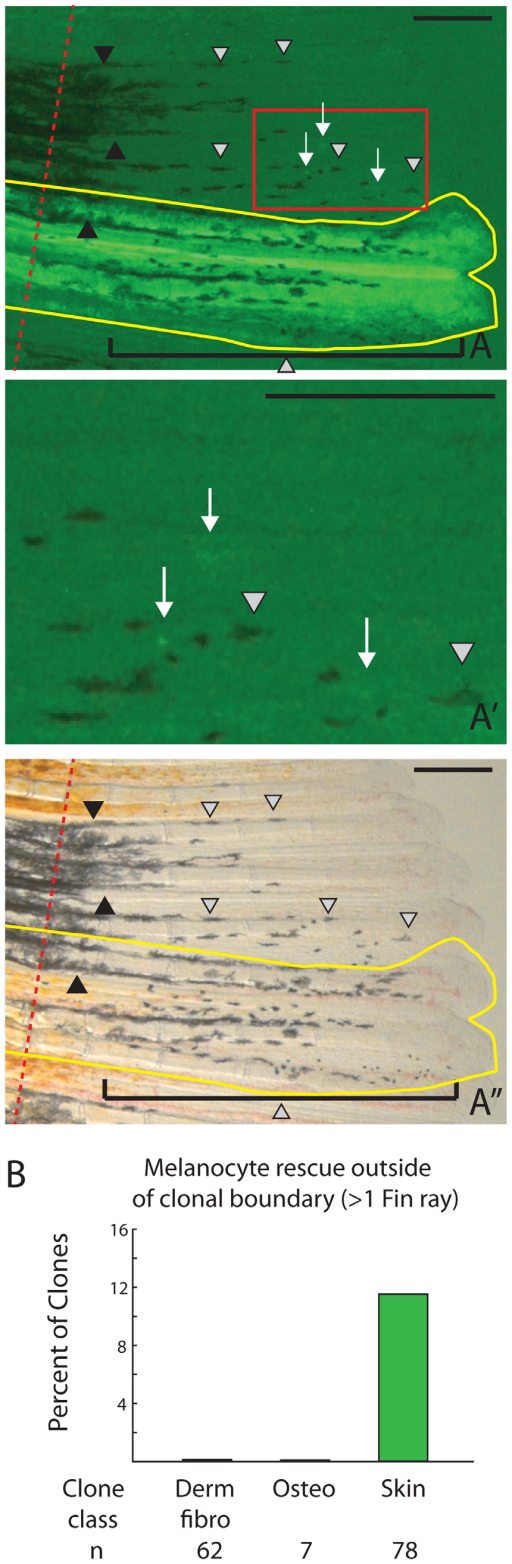
Melanocyte rescue occasionally occurs at a distance from the clone boundary in skin clones. A-A’’. While the majority of clones expressing *kitlga* only cause melanocyte rescue within the clone boundary (yellow line), some skin clones promote additional melanocyte rescue at a distance >1 fin ray (individual grey arrowheads. A’. Rogue labeled epidermal cells (arrows) along the edges of clonal boundaries may separate from the bulk of the clone mass and intercalate with unlabeled epidermal populations, supporting limited melanocyte regeneration at a distance. B. Quantification of the ability of different clone lineages to rescue melanocytes at significant distances from the clonal boundary. Brackets show the proximal-distal extent of melanocyte rescue within the clone. Black arrowheads indicate previously differentiated kit-independent melanocytes from the stump. Grey arrowheads indicate newly differentiated melanocytes. White arrows indicate GFP+ cells that are outside of the contiguous regenerated skin clone. Red dashed lines indicate the amputation plane. Scale bar  =  250 mm.

We also asked whether *kitlga* acts at a distance. It is not known if *kitlga* in the zebrafish is soluble and diffusible or only expressed as a membrane-bound form. In the mouse, one splice form in early development promotes melanocyte migration[30]. Later a second splice form produces a membrane-bound form that supports survival. Our first notion that *kitlga* is not expressed as a soluble peptide comes from never seeing rescue in the regenerating fin following heat shock in fish that contained body clones only (see above, Fig 4B). We next asked whether *kitlga* could rescue at closer distances to clones within the fin. When we looked at 164 clones of various cell lineages, the vast majority of them were only able to rescue melanocytes within the clone’s boundary. However, in a subset of skin clones (9/78, 11.5%) we could identify new *kit*-dependent melanocytes emerging at a distance of 1 fin ray or greater from the clonal boundary (Figure 7A,A’’). Although regenerating skin usually forms in a single contiguous patch, there is the possibility that a few labeled epidermal cells along the edges of a clone may intercalate with unlabeled epidermal cells (Fig 7A’), suggesting a smaller limit on the range of soluble kitlga. We saw no rescue of *kit*-dependent melanocytes at distances greater than 1 fin ray for any other clone type (Fig 7B). This suggests *kitlga* can rescue at short distances, raising the possibility that some protein is secreted rather than restricted to the membrane bound form.

## Discussion

Our goal was to develop a method to assess gene function in the context of regeneration *in vivo*. The use of the pt2-hsp70l vector, harboring both a lineage tracing and heat shock inducible cassette, allows us to assess rescue of cellular functions, in this case functional expression of the *kitlga* gene. Since *kitlga* acts non-autonomously on the melanocyte lineage, we could use this method to ask which cell lineages in the zebrafish fin are competent to support its expression, and consequently rescue melanocytes in the *kitlga* ^tc244b^ mutant background that lacks endogenous *kitlga* signaling. We found that *kitlga* could be functionally expressed by a variety of lineages, including dermal fibroblasts, skin, osteoblasts, vasculature, xanthophores and melanocytes, and resident blood. In contrast, neither lateral line nor iridiphore clones were able to support melanocyte rescue via the expression of *kitlga*. Efforts to show directly where *kitlga* expresses in the fin have failed. Our methodology allows us to test the functional consequence of gene expression from distinct lineages, although does not specifically address whether these lineages are the biological source of *kitlga* expression during fin regeneration.

The functional role of mammalian *kit ligand* (*Kitlg)* as it pertains to melanogenesis can be understood by considering both the form of protein produced and its site of expression. In mouse and human, 2 isoforms of *kitlg* mRNA have been detected, differing only in the alternative splicing of exon 6. This exon contains a proteolytic cleavage site that makes the extracellular portion of the *Kitlg* protein cleavable, and therefore able to act as a diffusible ligand. In mouse, the soluble isoform is expressed from the epithelial dermatome during early development, acting as a chemoattractant for neural crest derived melanoblasts [30]. Later the membrane-bound isoform of *Kitlg* is transiently expressed from dermal fibroblasts, and serves to promote melanoblast survival as they disperse throughout the body. Ultimately the melanocytes in the adult mouse localize to the hair follicles to provide hair pigmentation and are absent from the epidermis. In addition to follicular expression, humans also express *Kitlg* from keratinocytes to promote epidermal melanocyte homeostasis [31]. It is not known whether zebrafish *kitlga* has the same capacity to act as both a diffusible ligand and a membrane-bound supporter of melanocytes.

Our clonal *kitlga* functional expression analysis sheds light on possible roles for *kitlga* in zebrafish. In situ analysis of early developing zebrafish has shown expression of *kitlga* in the dorsal root ganglia, in the dorsal mytome, and throughout the skin [19]. These sites of *kitlga* expression are highly analogous to those seen in early mammalian development, as the neural crest-derived melanocytes are dispersed throughout the body. In contrast to mammalian *Kitlg*, zebrafish *kitlga* has only been detected as a single isoform. Sequence comparisons do not show any obvious similarity to the membrane cleavage site of the mammalian *kitlg* gene [19]. Consequently we wondered if expressing *kitlga* from each of the different lineages might reveal differential abilities of these lineages to provide *kitlga* function at a distance, which may suggest production of a diffusible factor.

Our findings show that during fin regeneration, *kitlga* predominantly acts at very short distances to support melanocyte rescue. First, in fish where *kitlga* clones were body-restricted, heat shock induced expression of *kitlga* failed to promote rescue of *kit*-dependent melanocytes in the regenerating fin. Secondly, in most clone types, we found no rescue of melanocytes at distances greater than 1 fin ray width from the margin of the clone (∼250 µm), suggesting that if *kitlga* is soluble, its activity rapidly decays as it diffuses from the expressing cell. Only for a small minority of *kitlga*-expressing skin clones (9/78 clone boundaries examined) did we find melanocyte rescue at more than 1 fin ray width from the clone, providing some evidence of action of *kitlga* at a distance. Since we have no antibodies to the zebrafish *kitlga*, we can speculate why this discrepancy is observed solely in skin clones. One attractive possibility is that skin has more abundant concentrations of protease and thus can generate some soluble *kitlga* protein.

Our analysis also showed evidence for some regional effects on melanocyte rescue relative to the lineage expressing *kitlga*. Dermal fibroblast clones always robustly promote *kit*-dependent melanocyte rescue. In contrast, many skin clones fail to rescue melanocytes. Analysis of the location of the skin clones within the fin revealed a strong bias to clones within presumptive melanocyte stripes providing rescue and clones within presumptive xanthophores stripes failing to rescue (p = 0.035). Typically during regeneration, *kit*-dependent melanocytes develop uniformly in both presumptive melanocyte and xanthophore stripe regions. After 7 dpa melanocytes begin to reorganize, persisting in the melanocyte stripes and migrating away from or dying in the xanthophore stripes [32]. This behavior is regulated by xanthophores. One possible explanation for this regional effect within skin clones is that inhibitory signals from the xanthophore stripe are blocking or degrading the *kitlga* signal when it is expressed from skin cells. Alternatively, inhibitory effects between xanthophores and melanocytes have been well documented [33]–[35], suggesting that a higher threshold of *kitlga* in the interstripe microenvironment may be required for melanocyte survival. Finally, skin in the xanthophores stripe may simply express low levels of *kitlga*, relative to skin clones in presumptive melanocyte stripes, leading to regional differences in melanocyte rescue.

## Supporting Information

Figure S1 **Variability of melanocyte regeneration from skin clones expressing** ***kitlga*** **.** A-A’. Representative skin clone categorized as strongly supporting melanocyte regeneration. B-B’. Representative skin clone categorized as weakly supporting melanocyte regeneration. C. Summary of the percentage of clonal area that contained melanocytes within skin clones. Thresholds were set using ImageJ to mask dark pixels (melanocytes) and the percentage of the clonal region masked was calculated. Averages and standard deviations for the two subgroups (strong and weak) are shown. Specific calculated percentages are shown for A’ and B’.
(TIF)
