# Supplementary figures and images for: Clonal Analysis of kit ligand a Functional Expression Reveals Lineage-Specific Competence to Promote Melanocyte Rescue in the Mutant Regenerating Caudal Fin
Source: PLoS One. 2014 Jul 10;9(7):e102317. doi: 10.1371/journal.pone.0102317 (PMC4092134; doi:10.1371/journal.pone.0102317)

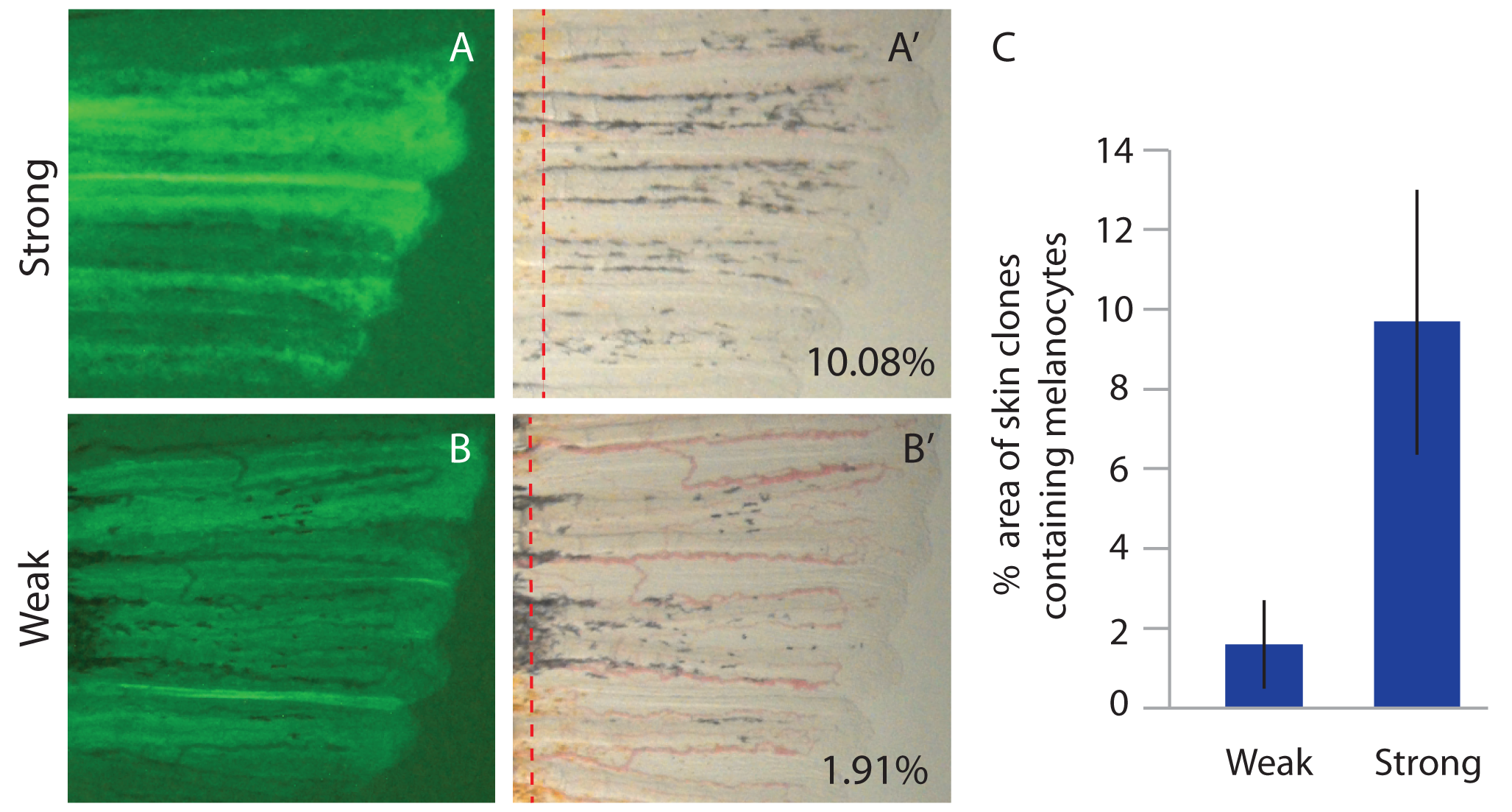

Supplement: Figure S1 — Variability of melanocyte regeneration from skin clones expressing kitlga . A-A’. Representative skin clone categorized as strongly supporting melanocyte regeneration. B-B’. Representative skin clone categorized as weakly supporting melanocyte regeneration. C. Summary of the percentage of clonal area that contained melanocytes within skin clones. Thresholds were set using ImageJ to mask dark pixels (melanocytes) and the percentage of the clonal region masked was calculated. Averages and standard deviations for the two subgroups (strong and weak) are shown. Specific calculated percentages are shown for A’ and B’. (TIF) [file pone.0102317.s001.tif]
